# Pediatric emergency department visits due to child abuse and neglect following COVID-19 public health emergency declaration in the Southeastern United States

**DOI:** 10.1186/s12887-021-02870-2

**Published:** 2021-09-13

**Authors:** Lindsey Rose Bullinger, Angela Boy, Stephen Messner, Shannon Self-Brown

**Affiliations:** 1 School of Public Policy, Georgia Institute of Technology, 685 Cherry St., 30332 Atlanta, GA USA; 2 Stephanie Blank Center for Safe and Healthy Children, Children’s Healthcare of Atlanta, Atlanta, USA; 3 School of Public Health, Georgia State University, Atlanta, USA

**Keywords:** child abuse, child neglect, pediatric emergency department, COVID-19

## Abstract

**Background:**

The ongoing worldwide COVID-19 pandemic has heightened several risk factors  for child abuse and neglect (CAN). We study whether COVID-19 and the public health response to it affected CAN-related pediatric emergency department (ED) visits in the southeastern United States (US).

**Methods:**

We performed a retrospective chart review on medical records of ED visits from a level I pediatric hospital system serving one of the largest metropolitan areas in the southeastern US from January through June 2018–2020. We used multivariate Poisson regression and linear regression to compare professionally identified CAN-related ED visits before and after a COVID-19 public health emergency declaration in 2020, relative to trends over the same period in 2018 and 2019.

**Results:**

Although the number of both overall pediatric ED visits and CAN-related ED visits declined, the number of CAN-related ED visits due to neglect from inadequate adult supervision increased by 62 % (*p* < 0.01). The number of CAN visits per 1,000 pediatric ED visits also increased by 97 % (*p* < 0.01). Finally, the proportion of CAN-related ED visits due to neglect from inadequate supervision increased by 100 % (*p* < 0.01).

**Conclusions:**

Physicians should be aware that patients who present with injuries during a pandemic may be victims of neglect due to changes in social structures in their households. In particular, maltreatment presenting to the ED shifted toward treating injuries and abuse resulting from inadequate supervision. Policymakers should consider the impacts of stay-at-home orders on child well-being when determining appropriate public health responses in the midst of a pandemic.

**Trial Registration:**

Not applicable.

**Supplementary Information:**

The online version contains supplementary material available at 10.1186/s12887-021-02870-2.

## Introduction

Social distancing measures implemented in the United States (US) have been necessary to slow the spread of COVID-19. But, public policies mandating staying-at-home and school closures [1], heightened parental stress related to pandemic impacts [2–4], and the retreat from services designed to support families in times of stress have been suspected to increase child abuse and neglect (CAN). Research has documented increases in several risk factors for CAN during the pandemic. For example, 911 calls reporting domestic violence have increased [5, 6], parents have reported deteriorating mental health, lower patience with their children, and heightened feelings of being overwhelmed by parenthood since before the pandemic [7, 8], and children’s behavioral health has declined since March 2020 [9]. Professionals of a CAN prevention program have also indicated increased CAN risk [10].

One challenge with documenting whether the pandemic has affected CAN, however, is measuring it. This is largely because children generally do not (or cannot) self-report CAN. Rather, identifying CAN victims often requires third party involvement. Common reporters include teachers, social workers, law enforcement, and medical personnel. Social distancing protocols have sharply diminished victims’ access to many mandated reporters and the resulting opportunity to be identified as at-risk and therefore reported to child protection services (CPS). For example, in various US states, compared to previous years reports of CAN to CPS [11–13], 911 calls reporting child abuse [5], and pediatric healthcare use [14, 15] are all down, offering fewer opportunities to intervene in suspected cases of CAN.

In contrast, less traditional data on CAN suggest otherwise with regard to various types of maltreatment. In particular, an increase in accidental injuries and ingestions has been reported in young children, suggesting inadequate adult supervision [16]. A US Level 1 trauma center reported an increase in the proportion of children with physical abuse-related injuries [17]. There are also suspicions of increased sexual abuse, but data are sparse. For example, Rape, Abuse, and Incest National Network, a US national sexual assault hotline, reported an increase in calls from minors since March 2020. Many (79 %) disclosed residing with a person who was abusing them during quarantine [18]. Additionally, social media data suggest an increase in violence directed toward children [19]. Finally, three studies use data from the Centers for Disease Control and Prevention’s National Syndromic Surveillance Program of US Emergency Department (ED) visits and the Pediatric Health Information System (PHIS) and found that both overall ED visits and CAN-related ED visits decreased during the pandemic [20–22]. However, the proportion of CAN-related ED visits significantly increased during the pandemic [21, 22]. Similar findings have emerged in other countries, such as England [23, 24], Singapore [25] and Italy [26].

In this study, we aim to answer three primary research questions:

1. What were the trends in the number of CAN-related ED visits before and after a southeastern US state (Georgia) issued a COVID-19 public health emergency declaration for 2020 relative to the same time periods in 2018 and 2019? Did these trends vary by maltreatment type, child characteristics, or severity of CAN?
2. How did trends in CAN-related ED visits—overall, by maltreatment type, by child characteristics, and by severity—before and after a COVID-19 public health emergency declaration compare to trends in overall pediatric ED visits?
3. Relative to trends in overall CAN-related ED visits before and after a COVID-19 public health emergency declaration, were there shifts in the types of maltreatment, severity of CAN, or child characteristics that presented to the ED?

We hypothesize that as compared to 2018 and 2019, in 2020 the number of CAN-related ED visits declined, due to documented reductions in overall pediatric ED use during the height of the early stages of the pandemic. However, we predict that in 2020 CAN-related visits as compared to overall pediatrics ED visits increased from 2018 to 2019 due to more CAN risk factors present during the pandemic. Finally, we predict a shift in the type of CAN that presented to the ED to be more toward injuries due to inadequate adult supervision—due to widespread and long-lasting school closures—and away from injuries from physical abuse, which often requires third party detection.

To answer these questions, we performed a comprehensive analysis using medical records data from a large, urban pediatric hospital network that serves the entire state of Georgia, and is the only free-standing pediatric healthcare system and pediatric Level 1 Trauma Center in the state.

## Study data and methods

This retrospective study relied on data from patient chart and medical record reviews from the Children’s Healthcare of Atlanta (Children’s) system (Atlanta, GA, US) from the first 26 weeks of 2018, 2019, and 2020. We included patients if they were under age 18, admitted to the emergency departments (ED) of one of the three hospitals that make up the Children’s system, and CAN was identified by a professional during an exam. Cases were identified from three sources: (1) social work referrals for abuse, (2) ICD-10 and Pediatric Emergency Care Applied Research Network (PECARN) coding in the electronic medical record (EMR), and (3) cases identified at weekly trauma rounds. Clinicians refer patients to social work when abuse or neglect is suspected. Social workers then make a referral to the state’s Division of Family and Children’s Services (DFCS) and document the referral in the EMR. Cases were also identified by reviewing all cases with a final diagnosis (ICD-10) or PECARN diagnosis of any type of child abuse or neglect. These cases are reported monthly to the child advocacy center (CAC). Finally, cases were identified in weekly trauma rounds that are attended by Children’s trauma clinicians. We removed duplicate cases. During the first 26 weeks of 2018 and 2019, 239,437 children visited Children’s EDs; 2,296 of these (0.95 %) were CAN cases. In 2020, 83,491 patients visited Children’s EDs; of these visits in 2020, 1,160 (1.4 %) were CAN cases.

The Children’s system serves patients from all 159 Georgia counties and treats over 500,000 children per year in over 1 million patient visits. Over 50 % of the patient population is publicly insured. This study was approved by both Children’s Healthcare of Atlanta and Georgia Institute of Technology IRBs.

### Measures

The primary outcomes are (1) the number of visits admitted through the ED that involved CAN; (2) the rate of ED visits that were CAN visits; and (3) the proportion of CAN visits that were identified as a specific maltreatment type along with child characteristics (e.g., age and sex). The first measure is the raw number of pediatric ED visits where a professional suspected CAN, as measured by chart review, regardless of the chief concern for the visit. The second measure is the number of visits that were identified as CAN divided by the total number of ED visits (per 1,000 visits). This measure indicates whether there was a shift in the prominence of CAN cases in the ED during the pandemic. The final outcome measure is the number of ED visits that consist of different types of maltreatment (any form of neglect, neglect due to a lack of supervision, medical neglect, physical abuse, and sexual abuse), a measure of acuity (whether the CAN visit resulted in time spent in the pediatric intensive care unit (PICU)), and child characteristics (ages 0–1, 2–5, 6–10, 11–17, female, male, non-Hispanic white, non-Hispanic Black, Asian, or Hispanic), divided by the total number of CAN-related visits. In other words, this measure is the percent of all CAN-visits that consist of various maltreatment types, severity of CAN, or specific child characteristics. Child characteristics are parent-reported. Race/ethnicity is often reported in a non-standard manner and is missing for approximately 6 % of cases; therefore, we are cautious in our interpretation of the differences across race/ethnicity. When performing the age, sex, and race/ethnicity-specific analyses, the denominators of ED visits are limited to ED visits among those age groups, sexes, and races/ethnicities. The maltreatment type is designated by the social worker who responds to the case, and are not mutually exclusive; for example, a visit may be labeled both physical abuse and neglect due to lack of supervision.

### Statistical analysis

We computed daily visit counts, rates, and proportions, stratified by maltreatment type, acuity, and child characteristics. We limit the analysis to the same time period (weeks 1–26) of 2018, 2019, and 2020. We do this due to potential seasonality in CAN perpetration. Georgia’s Governor declared a public health emergency on March 14, 2020 and ordered schools to close beginning March 18, 2020, the 10th week of the year. Previous research has documented this time period as the most sensitive for people beginning social distancing and staying home nearly exclusively [13, 27–29]. Therefore, we split the study period into weeks 1–10 and weeks 11–26.

We use multivariate linear regression—estimated using Poisson for the number of visits and ordinary least squares for the rate/proportion—to compare the CAN visit outcomes before and after the 10th week of 2020 relative to those over the same period from 2018 to 2019, thereby estimating the effects of this bundle of pandemic-related policies. The unit of analysis is the date, across January 1 through June 30 (the first 26 weeks) of 2018, 2019, and 2020, totaling 543 days. We adjust for (1) seasonality in trends in visits by including binary variables indicating the week of the year, (2) baseline differences in 2018 and 2019 relative to 2020 by including a binary variable for each year, and (3) the potential differential effect of weekend days by including indicator variables for the day-of-the-week.

## Results

Appendix A reports sample means in the outcome variables, stratified by time period. On average, Children’s facilities experienced about 729 visits to their emergency departments per day between January and June 2018–2019. About 6 of these visits were identified by a professional to be CAN. During weeks 1–10 of 2020, daily ED visits were around 693, with about 7 of them related to CAN. Overall, weeks 11–26 of 2020 saw fewer pediatric ED visits and fewer CAN-related ED visits than all of 2018–2019 and the first 10 weeks of 2020. Weekly trends of these two variables are shown in Appendix B. These trends are consistent with previously documented trends in overall pediatric ED visits and CAN-related ED visits [20–22].

**Fig. 1:**
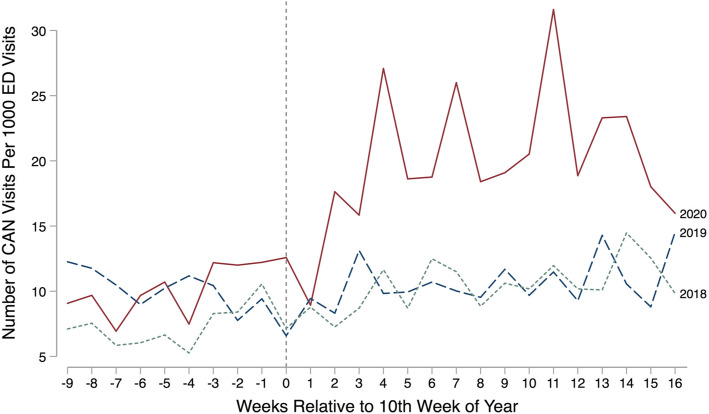
Total CAN ED Visits Relative to Total Pediatric ED Visits. Source: Children’s medical records January through June 2018, 2019, and 2020. Notes: Outcome is the number of CAN-related ED visits divided by the total number of pediatric ED visits. The vertical line represents the week of the state’s COVID-19 public health emergency declaration and school closure mandate in 2020

In contrast, relative to the number of ED visits, the number of CAN-related visits increased in weeks 11–26 of 2020. Weekly trends are documented in Fig. 1.

Table 1 shows the main results from the adjusted regression analysis. Column 1 reports the mean CAN outcome during the first 10 weeks of each year (2018–2019 versus 2020), which was before the state’s emergency declaration went into effect in 2020. Column 2 reports the mean CAN outcome for weeks 11–26 (after the 2020 emergency declaration) of each year. Column 3 shows the difference within year before and after the week of the emergency declaration. Column 4 shows the difference between rows 1 and 2 in column 3, while adjusting for binary variables indicating year, week, and day-of-the-week. Column 4 is the parameter of interest, documenting the effect of the COVID-19 emergency declaration in Georgia. Table 1 reports the findings for overall CAN visits, and visits due to neglect from lack of supervision, while Appendix C produces the estimate in Column 4 for all CAN outcomes by maltreatment type, child characteristics, and severity of maltreatment.

**Table 1:** Effect of Georgia’s Emergency Declaration on CAN Visits to Children’s EDs

	(1)	(2)	(3)	(4)
	Mean Weeks 1–10	Mean Weeks 11–26	Difference (Column 1 - Column 2)	Adjusted Difference (Row 1-Row 2 + Year, Week, & Day-of-Week Dummy Variables)
**Panel A: Number of Children Visiting Children’s Facilities with Suspected CAN**				
2020	7.03	5.94	-1.09***	
2018–2019	6.20	6.46	0.26	
Difference (Column 4)				-0.21***
				(0.07)
**Panel B: Number of Children Visiting Children’s Facilities due to Neglect from Lack of Supervision**				
2020	0.62	1.24	0.62***	
2018–2019	0.68	0.73	0.05	
Difference (Column 4)				0.62***
				(0.19)
**Panel C: CAN ED Visits Relative to All ED Visits**				
2020	10.21	20.43	10.23***	
2018–2019	8.75	10.53	1.78***	
Difference (Column 4)				8.47***
				(1.01)
**Panel D: Neglect from Lack of Supervision ED Visits Relative to All ED Visits**				
2020	0.87	4.28	3.40***	
2018–2019	0.97	1.19	0.21	
Difference (Column 4)				3.17***
				(0.35)
**Panel E: Neglect from Lack of Supervision ED Visits Relative to CAN ED visits**				
2020	0.10	0.22	0.12***	
2018–2019	0.11	0.12	0.01	
Difference (Column 4)				0.11***
				(0.03)

**Source:** Children’s Healthcare of Atlanta data weeks 1–26 of 2018, 2019, and 2020. **Notes:** The unit of analysis is date. *N* = 181 days in each of 3 years = 543 for each cell. The outcomes are: the number of children visiting Children’s facilities’ EDs with confirmed child abuse or neglect (Panels A-C), the rate of child abuse and neglect visits relative to all ED visits, per 1,000 visits (Panels D-F), and the percent of all CAN ED visits due to specific maltreatment types or child characteristics (Panels G-H). Adjusted regressions include week, year, and day of the week fixed effects. Panels A-B estimated as a Poisson model. Panels C-E are estimated as OLS. Robust standard errors are in parentheses

*p < 0.10, **p < 0.05, ***p < 0.01

Panel A of Table 1 shows that during weeks 11–26 in 2020 there were 1.09 fewer CAN-related ED visits per day than the first ten weeks of the year (*p* < 0.01). In contrast, there was no significant difference in the number of CAN-related visits during this time period in 2018–2019. After adjusting for year, week, and day-of-the-week indicator variables, Column 4 shows that CAN-related ED visits declined by about 21 % (*p* < 0.01) following the state of Georgia’s emergency declaration.

Panel B shows that the number of children visiting Children’s facilities who were neglected due to lack of supervision increased over the course of 2020 by 0.62 visits per day (*p* < 0.01). In 2018–2019, there was no significant difference in the number of visits for neglect due to lack of supervision. Despite the overall decrease in CAN-related ED visits, Column 4 shows an *increase* in the number of children visiting Children’s EDs for neglect due to lack of supervision by about 62 % (*p* < 0.01). These visits consist of pediatric injuries due to inadequate adult supervision, or child abuse that resulted from a lack of supervision, including injuries that were unwitnessed, ingestions (unintentional or intentional), drownings or near drownings, falls, or gunshot wounds. Appendix C reports additional outcomes, showing that the overall decrease is driven by EDs seeing fewer cases of physical abuse, adolescent children (aged 11–17), male children, and non-Hispanic white children.

Panels C and D show the number of CAN-related visits per 1,000 ED visits. As seen in Panel C, the number of CAN-related visits per 1,000 ED visits increased by 10.23 visits in 2020 (*p* < 0.01), but only 1.78 visits during the same time period in 2018–2019. The fully adjusted regression shows an increase of 8.47 CAN-related visits per 1,000 ED visits (*p* < 0.01). Relative to the 2018–2019 mean of 8.75, this change represents about a 97 % increase. Panel D shows a similar pattern: visits from neglect due to lack of supervision increased by 3.17 per 1,000 ED visits (*p* < 0.01), or about 327 %. Other large relative increases in the proportion of CAN-related ED visits were among children aged 5 or under, females, non-Hispanic black children, and child victims of sexual abuse (Appendix C).

**Fig. 2:**
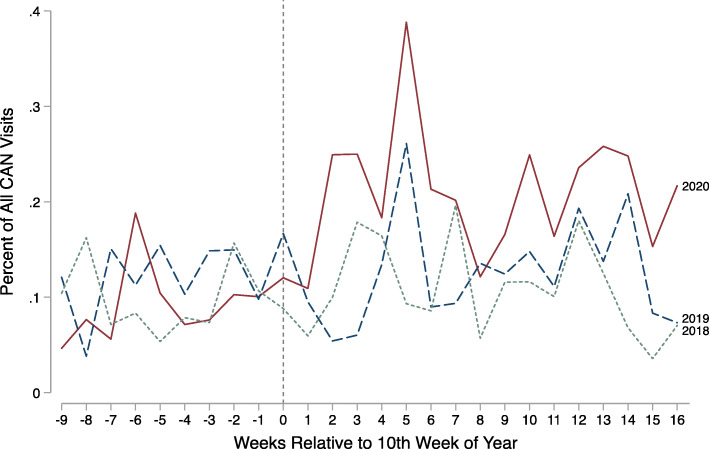
Neglect Due to Inadequate Supervision ED Visits Relative to Total CAN Visits. Source: Children’s medical records January through June 2018, 2019, and 2020. Notes: Outcome is the number of CAN-related ED visits due to neglect from inadequate supervision divided by the number of CAN-related ED visits. The vertical line represents the week of the state’s COVID-19 public health emergency declaration and school closure mandate in 2020

Finally, Panel E shows the proportion of CAN-related ED visits that are due to specific maltreatment types. This figure documents a substantial increase in the percent of CAN-related visits that are due to neglect from inadequate supervision. In particular, visits to the ED due to inadequate adult supervision increased by 0.11 visits per ED visit (*p* < 0.01), doubling the percent of CAN-related visits due to inadequate supervision. Figure 2 shows weekly trends in the percent of CAN-related ED visits due to a lack of supervision. There was no significant change in the percent of CAN-related ED visits in other maltreatment types or child characteristics, with one exception: the percent of all CAN-related ED visits that occurred among children ages 0–1 increased by about 33 % (Appendix C).

## Discussion

Early in the pandemic, physicians, child advocates, and researchers speculated that CAN was on the rise [30]. But, various forms of data, including CAN reports to child welfare agencies, 911 calls and police reports, and social media accounts provide mixed evidence [5, 11–13, 19]. In this paper, we document trends in CAN-related ED visits by performing a deep review of one large, urban healthcare network’s medical records system, to explore nuances in CAN trends that have been overlooked by previous research.

We present two main findings. First, despite overall pediatric ED visits and CAN-related ED visits declining during the pandemic, the number of ED visits from neglect due to lack of supervision actually *increased *by about 62 % (*p* < 0.01). This finding is important since so many other sources of data consistently report declines in the raw number of visits [20–22]. This finding is also consistent with reports from the National Poison Data System that calls to poison centers regarding ingestions and inhalation increased in 2020 relative to the same period in 2018–2019 [16], particularly among children aged 5 and under. Other research has shown that fractures and injuries occurring in the home, such as bicycle injuries, have increased [25, 31].

Second, as a percent of all CAN-related ED visits, visits for neglect due to lack of supervision doubled, indicating a shift in the types of CAN presenting to the ED. This increase may be due to a decline in all other types of CAN-related ED visits (e.g., physical and sexual abuse). However, coupled with the finding that the raw number of visits due to lack of supervision actually increased, we assert that the nature of visits to the ED shifted almost completely toward treating injuries and abuse due to inadequate supervision. When paired with both the literature on CPS reports and pediatric ED use, this study suggests that CAN is still occurring but is likely being under-reported and under-detected.

In addition, we find that the largest relative increases in the proportion of CAN-related ED visits were among children aged 5 or under and females. The greatest increases in the proportion of CAN type were for sexual abuse and neglect due to lack of supervision. Although limited evidence exists, sexual assault is postulated to have increased during the pandemic—as often occurs during states of emergency—and risk for minors has increased with more time at home with potential perpetrators [32]. Similarly, prior research documents that more time spent at home during the COVID-19 pandemic has been associated with more referrals of supervisory neglect [13].

Appropriate public policy responses differ based on the type of CAN that children experience [33, 34]. For example, existing evidence-based CAN prevention programs typically focus on specific CAN types, with effective programs targeting parent perpetration risk for child physical abuse and neglect [35], and effective programs for sexual abuse offering community-based training for adults [36] or school-based for children [37]. We find that neglect due to lack of supervision increased after schools closed and people were instructed to stay at home nearly exclusively. This finding points to the need for increased resources to support parents who continue to work outside the home without access to childcare or in-person schooling and those who have transitioned to working from home and may be experiencing challenges juggling the multiple responsibilities of work and parenting demands.

The results of our study highlight the need for societal measures that focus on minimizing situations where children are inadequately supervised and also increase their exposure to mandated reporters during a pandemic. ED physicians, in particular, should have a heightened awareness of the likelihood that patients who present with injuries during a pandemic may be victims of neglect due to inadequate adult supervision that result from changes in the social structures of their households. Inquiring about the changes in daycare, school, and presence of adult caregivers should become a routine part of the history when a child presents to the ED with an injury, particularly during a pandemic and other large-scale disasters and crises. The decrease or loss of adult supervision can lead to not only physical injuries as a result of exposure to dangerous situations, but also increases the opportunity for other forms of abuse, particularly sexual abuse. Primary care physicians should also make every attempt to continue to safely see children for their well visits during a pandemic in order to increase the exposure of children to mandated reporters. Inquiries should be made when children have repeated cancelations or no shows to scheduled visits, and reports should be made to CPS in cases of medical neglect. By continuing to keep these safety measures in place during a pandemic, children will continue to benefit from having mandated reporters advocating for their well-being.

### Limitations

This study is limited to one hospital system in the southeastern United States. Given the consistencies of the declines in overall pediatric ED use and CAN-related ED use to studies using data from multiple hospital systems [20–22, 25, 26], however, we expect our results are likely representative of large, urban hospital systems and their patient populations.

Our study is also limited to the first 3 months of the pandemic. Estimating longer-term effects may show different patterns, as families may have adjusted to the “new normal” or may have experienced compounding hardships over time.

Despite these limitations, the nuance this study adds to the burgeoning literature documenting trends in CAN during the COVID-19 pandemic is valuable. Our findings suggest that researchers should continue to use creative sources of data to measure CAN and dig deeper into them to paint a full picture of CAN during COVID-19.

## Conclusions

More children left unattended and at-risk for CAN has been an unintended and negative consequence of the public health response to the pandemic. Policymakers should increase outreach to families who may be at risk—including those who have not historically been considered at risk but whose challenges may increase rapidly during pandemic and disaster circumstances—and provide emergency social and economic supports [34]. Healthcare professionals working in areas with social distancing may consider extended durations of time at home an additional risk factor for CAN, and should increase contact with families throughout the crisis . Recommendations for how to ensure contact and safety assessments can be conducted through virtual appointments may be necessary to ensure such healthcare professionals can implement best practices in times where physical contact are significantly restricted. Finally, the results of this study are important when considering the long-term consequences of childhood abuse and neglect.

## Supplementary information

**Additional file 1.**

## Data Availability

The data that support the findings of this study are available from Children’s Healthcare of Atlanta but restrictions apply to the availability of these data, which were used under license for the current study, and so are not publicly available. Data are however available from the authors upon reasonable request and with permission of Children’s Healthcare of Atlanta.

## Abbreviations

CAN: child abuse and neglect
ED: emergency department
CPS: child protective services
Children’s: Children’s Healthcare of Atlanta
PICU: pediatric intensive care unit
PECARN: Pediatric Emergency Care Applied Research Network
EMR: electronic medical record
CAC: child advocacy center
