# Supplementary material for: Pediatric emergency department visits due to child abuse and neglect following COVID-19 public health emergency declaration in the Southeastern United States
Source: BMC Pediatr. 2021 Sep 13;21:401. doi: 10.1186/s12887-021-02870-2 (PMC8435405; doi:10.1186/s12887-021-02870-2)
Supplement: Supplementary file 1 — Additional file 1. [file 12887_2021_2870_MOESM1_ESM.pdf]

Appendix A. Summary Statistics by Time Period

	2018-2019		2020	
	Weeks 1-10	Weeks 11-26	Weeks 1-10	Weeks 11-26
**Panel A: Number of Children Visiting Children's Facilities with Suspected Child Abuse or Neglect**				
Total ED Visits	729.11	622.03	693.49	312.03
Total CAN ED Visits	6.09	6.53	7.06	5.95
All Neglect	1.78	2.20	2.51	2.38
Neglect from Inadequate Supervision	0.68	0.73	0.61	1.22
Medical Neglect	0.23	0.23	0.21	0.13
Physical Abuse	1.83	1.68	2.03	1.36
Sexual Abuse	1.68	1.76	1.77	1.59
Aged 0-1	1.23	1.10	1.26	1.02
Aged 2-5	1.45	1.62	1.46	1.39
Aged 6-10	1.03	1.12	1.20	0.96
Aged 11-17	2.02	2.20	2.71	2.11
Female	3.48	3.68	3.83	3.46
Male	2.61	2.84	3.20	2.48
Non-Hispanic White	1.19	1.40	1.51	1.27
Non-Hispanic Black	3.99	4.15	4.51	3.77
Asian	0.04	0.09	0.03	0.06
Hispanic	0.63	0.67	0.77	0.57
PICU	0.68	0.78	0.49	0.72
**Panel B: CAN ED visits relative to all ED visits**				
Total CAN Visits	8.61	10.58	10.25	20.13
All Neglect	2.55	3.55	3.69	8.06
Neglect from Inadequate Supervision	0.97	1.18	0.88	4.18
Medical Neglect	0.31	0.38	0.31	0.45
Physical Abuse	2.62	2.69	2.94	4.65
Sexual Abuse	2.33	2.87	2.59	5.33
Aged 0-1	6.21	6.37	6.78	13.58
Aged 2-5	7.62	9.84	7.57	20.82
Aged 6-10	7.04	8.86	8.72	15.16
Aged 11-17	12.29	14.97	15.76	24.19
Female	10.18	12.57	11.41	24.22
Male	7.17	8.74	9.08	16.10
Non-Hispanic White	8.27	10.83	11.47	15.73
Non-Hispanic Black	10.52	12.47	12.10	26.55
Asian	2.00	4.80	1.79	7.72
Hispanic	4.59	6.04	5.62	11.63
PICU	0.96	1.25	0.69	2.47
**Panel C: Maltreatment-specific visits relative to CAN ED visits**				
All Neglect	0.29	0.34	0.35	0.41
Neglect from Inadequate Supervision	0.11	0.12	0.09	0.21
Medical Neglect	0.04	0.03	0.02	0.02
Physical Abuse	0.32	0.26	0.30	0.23
Sexual Abuse	0.26	0.26	0.26	0.26
Aged 0-1	0.21	0.17	0.16	0.18
Aged 2-5	0.24	0.25	0.20	0.23
Aged 6-10	0.16	0.16	0.17	0.15
Aged 11-17	0.32	0.34	0.40	0.36
Female	0.57	0.56	0.55	0.57
Male	0.43	0.44	0.45	0.43
Non-Hispanic White	0.19	0.22	0.21	0.22
Non-Hispanic Black	0.66	0.62	0.64	0.62
Asian	0.01	0.01	0.01	0.01
Hispanic	0.11	0.10	0.11	0.10
PICU	0.12	0.13	0.08	0.13
N	139	222	70	112

Appendix B. Trends in Number of ED Visits (Top Panel) and Number of CAN-related ED Visits (Bottom Panel)

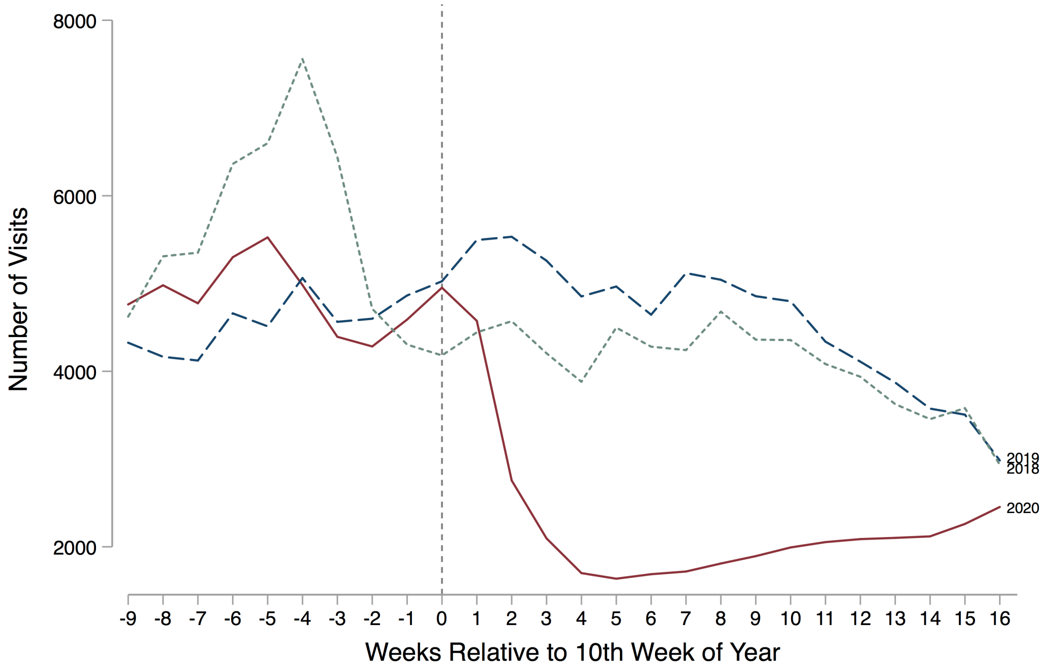

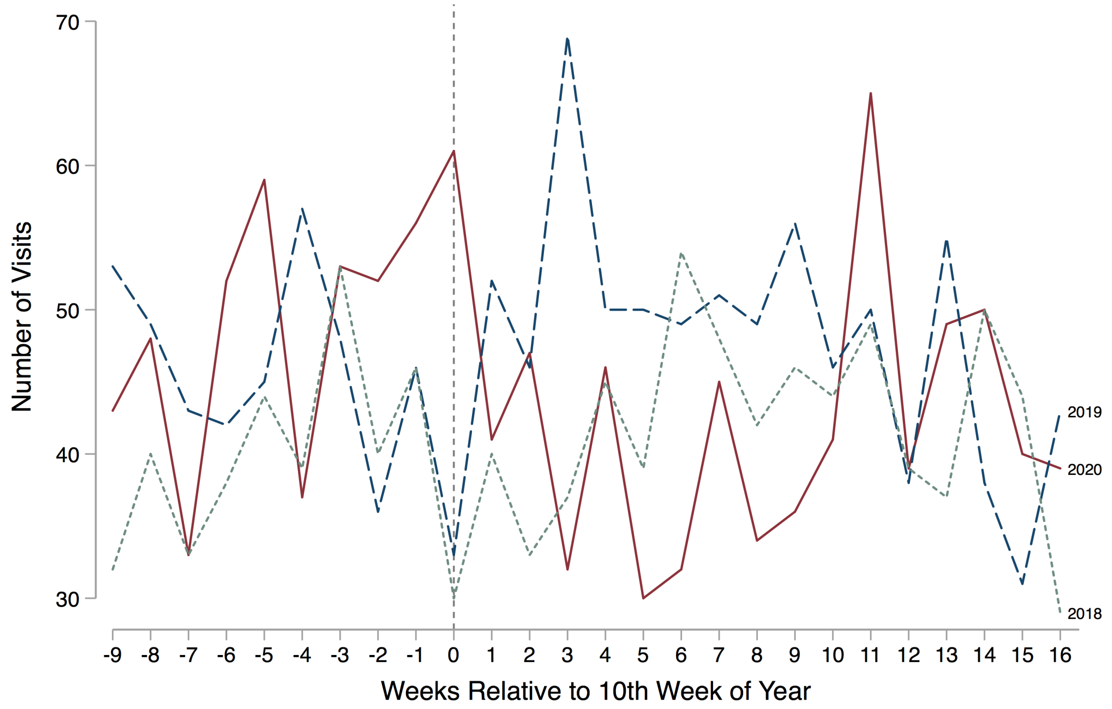

Appendix C. Regression Results For All Outcomes

	Total CAN Visits	All Neglect	Neglect from Inadequate Supervision	Medical Neglect	Physical Abuse	Sexual Abuse	Aged 0-1	Aged 2-5	Aged 6-10	Aged 11-17	Female	Male	Non-Hispanic White	Non-Hispanic Black	Asian	Hispanic	PICU
**Panel A: Number of Children Visiting CHOA facilities with Suspected Child Abuse or Neglect**																	
Post Emergency Declaration	-0.21***	-0.22*	0.62***	-0.55	-0.27**	-0.12	-0.08	-0.12	-0.31*	-0.29**	-0.10	-0.34***	-0.30*	-0.18*	-0.43	-0.34	0.16
	(0.07)	(0.13)	(0.19)	(0.42)	(0.14)	(0.15)	(0.17)	(0.16)	(0.18)	(0.13)	(0.10)	(0.11)	(0.16)	(0.10)	(0.80)	(0.21)	(0.23)
Mean Y in 2018-2019	6.20	1.83	0.68	0.24	1.87	1.71	1.24	1.45	1.08	2.07	3.58	2.62	1.22	4.08	0.03	0.63	0.67
**Panel B: CAN ED visits relative to all ED visits**																	
Post Emergency Declaration	8.47***	3.63***	3.17***	0.07	1.79***	2.38***	7.03***	11.72***	4.75**	6.37***	11.28***	5.73***	2.20	13.26***	2.41	4.53***	1.45***
	(1.01)	(0.59)	(0.35)	(0.16)	(0.52)	(0.57)	(1.50)	(2.01)	(2.06)	(2.20)	(1.69)	(1.16)	(1.93)	(1.74)	(3.64)	(1.72)	(0.32)
Mean Y in 2018-2019	8.75	2.61	0.97	0.32	2.65	2.37	6.25	7.60	7.31	12.46	10.44	7.20	8.47	10.69	1.94	4.59	0.95
Relative % Change	96.8%	139.1%	326.8%	21.9%	67.5%	100.4%	112.5%	154.2%	65.0%	51.1%	108.0%	79.6%	26.0%	124.0%	124.2%	98.7%	152.6%
**Panel C: Maltreatment-specific visits relative to CAN ED visits**																	
Post Emergency Declaration	--	0.02	0.11***	0.00	0.00	0.00	0.07**	0.02	-0.03	-0.06	0.04	-0.04	-0.02	0.01	0.00	-0.00	0.03
	--	(0.04)	(0.03)	(0.01)	(0.04)	(0.04)	(0.03)	(0.03)	(0.03)	(0.04)	(0.04)	(0.04)	(0.04)	(0.04)	(0.01)	(0.03)	(0.03)
Mean Y in 2018-2019	--	0.30	0.11	0.04	0.32	0.26	0.21	0.24	0.17	0.32	0.57	0.43	0.19	0.66	0.01	0.11	0.12
Relative % Change	--	6.7%	100.0%	0.0%	0.0%	0.0%	33.3%	8.3%	-17.6%	-18.8%	7.0%	-9.3%	-10.5%	1.5%	0.0%	0.0%	25.0%

Source: Children's Healthcare of Atlanta data weeks 1-26 of 2018, 2019, and 2020. Notes: The unit of analysis is date. N=181 days in each of 3 years =543 for each cell. Each cell represents the estimate from column 4 in Table 1. The outcomes are: the number of children visiting CHOA facilities' EDs with confirmed child abuse or neglect (Panel A), the rate of child abuse and neglect visits relative to all ED visits, per 1,000 visits (Panel B), and the percent of all CAN ED visits due to specific maltreatment types or child characteristics (Panel C). In Panel B, the denominator is age and gender-specific. Post Emergency Declaration=1 for weeks 11-26 of 2020. Regressions include week, year, and day of the week fixed effects. Panel A estimated as a Poisson model. Panels B and C are estimated as OLS. *p<0.10, **p<0.05, ***p<0.01.
